# The effect of a sequential structure of practice for the training of perceptual-cognitive skills in tennis

**DOI:** 10.1371/journal.pone.0174311

**Published:** 2017-03-29

**Authors:** David P. Broadbent, Paul R. Ford, Dominic A. O’Hara, A. Mark Williams, Joe Causer

**Affiliations:** 1 Research Institute for Sport and Exercise Sciences, Liverpool John Moores University, Liverpool, United Kingdom; 2 Centre for Cognitive Neuroscience, Brunel University, London, United Kingdom; 3 Centre for Sport and Exercise Science and Medicine, University of Brighton, Brighton, United Kingdom; 4 Department of Health, Kinesiology, and Recreation, University of Utah, Salt Lake City, Utah, United States of America; Universidade de Tras-os-Montes e Alto Douro, PORTUGAL

## Abstract

**Objective:**

Anticipation of opponent actions, through the use of advanced (i.e., pre-event) kinematic information, can be trained using video-based temporal occlusion. Typically, this involves isolated opponent skills/shots presented as trials in a random order. However, two different areas of research concerning representative task design and contextual (non-kinematic) information, suggest this structure of practice restricts expert performance. The aim of this study was to examine the effect of a sequential structure of practice during video-based training of anticipatory behavior in tennis, as well as the transfer of these skills to the performance environment.

**Methods:**

In a pre-practice-retention-transfer design, participants viewed life-sized video of tennis rallies across practice in either a sequential order (sequential group), in which participants were exposed to opponent skills/shots in the order they occur in the sport, or a non-sequential (non-sequential group) random order.

**Results:**

In the video-based retention test, the sequential group was significantly more accurate in their anticipatory judgments when the retention condition replicated the sequential structure compared to the non-sequential group. In the non-sequential retention condition, the non-sequential group was more accurate than the sequential group. In the field-based transfer test, overall decision time was significantly faster in the sequential group compared to the non-sequential group.

**Conclusion:**

Findings highlight the benefits of a sequential structure of practice for the transfer of anticipatory behavior in tennis. We discuss the role of contextual information, and the importance of representative task design, for the testing and training of perceptual-cognitive skills in sport.

## Introduction

Performance in most sports involves the execution of perceptual-cognitive and motor skills to affect the current environmental situation [1]. Perceptual-cognitive skill involves the ability to use vision to locate and identify key environmental information so as to process and integrate it with existing knowledge in order to make *judgments* to predict opponent and teammate behavior (anticipation) and to select and execute appropriate actions (decision making) [2]. Researchers examining these skills often use video-based tasks that are occluded at key time points (e.g., opponents’ ball-racket contact). The majority of this research has focused on testing and training the use of advanced postural information to anticipate the outcome of isolated opponent actions using video-based tasks [3]. These tasks attempt to recreate conditions experienced when performing in the sport, so as to promote transfer of learning from the task to performance environment, while maintaining experimental control (for a review, see [4]). Using these methods, researchers have shown that experts across multiple sports are superior at anticipating opponent actions compared to less-skilled counterparts. Moreover, anticipation can be improved using video-based training, with researchers demonstrating transfer from this type of training to field-based settings [5]. However, concerns have been raised regarding the experimental designs employed [6] including the absence of contextual information in these tasks [7]. A suggestion is that this approach may be restricting the advancement of knowledge and the application of findings to the performance environment. In the current paper, we seek to address some of these limitations by examining the effect of practice structure (and a manipulation of contextual information) on the transfer of anticipation in tennis from a video-based training task to a field-based setting.

The design of video-based experimental tasks used to examine perceptual-cognitive skills enables between-participant repetition of key information, providing greater experimental control. However, this approach has been criticized for lacking action fidelity and task functionality [6]. Action fidelity refers to the response of the performer remaining consistent between the performance environment and the experimental task [8]. Task functionality refers to whether the constraints a performer is exposed to, and must act upon, in the experimental task are the same as those that they will be exposed to in the performance environment [9]. Tasks used in previous research examining perceptual-cognitive skills have been criticized for using simplistic responses that lack action fidelity, such as verbal and button press responses, to small and static visual displays that lack task functionality [10]. Action fidelity and task functionality increase specificity of practice [11], which holds that skills and their underlying processes are specific to the conditions in which they are acquired, such that changing those conditions to be less specific negatively affects their acquisition.

Some researchers have attempted to increase action fidelity and task functionality when examining perceptual-cognitive skills. Dicks, Button, and Davids [12] compared five experimental conditions to investigate anticipation of soccer penalty kicks. They used two life-sized video conditions filmed from the perspective of the goalkeeper with either a verbal or joystick response. Additionally, they used three *in situ* conditions involving either a verbal response, a simplified movement (side step) or a complex interceptive movement response to kicks as per the performance environment (save). Responses were more accurate in the *in situ* movement conditions compared to the video-based and non-movement conditions, providing support for increasing action fidelity and task functionality. However, the difficulty of increasing action fidelity and task functionality while maintaining experimental control was clearly demonstrated in this study. In the video-based conditions, ball flight was occluded after its initial portion, whereas in the in situ conditions full ball flight was available. Moreover, the in-situ interception condition had two possible outcomes for penalty kicks, whereas all other conditions had six options. Both of these methodological limitations affect response accuracy in favor of in situ conditions. In addition, despite the researchers attempts to control between-participant conditions in situ, variability existed within the run-up times and ball flight of the kicks between participants and when compared to the video-based conditions.

Other researchers have attempted to increase action fidelity and task functionality by using life-sized dynamic videos filmed from the perspective of the athlete [13]. They have used complex, rather than simple, responses that are similar to the performance environment, albeit video-based tasks are limited in terms of action fidelity because they cannot usually be combined with interceptive actions. Findings from studies using these methods have shown transfer of learning to the performance environment (for example, see [14–17]), indicating appropriate action fidelity and task functionality [18].

Another criticism of the video-based temporal occlusion tasks is the focus on the use of advanced postural information such that important contextual information normally found in the performance environment is considerably reduced [7]. Cañal-Bruland and Mann [7] highlighted the need for researchers to consider “the influence of broader situational or contextual (non-kinematic) sources of information” (p. 1). These sources of information were highlighted as important in research conducted by Triolet, Benguigui, Le Runigo, and Williams [19] who quantified anticipation in elite level tennis matches. They found that anticipatory behavior occurs more when in an unfavorable/defensive situation and that a proportion of these anticipatory movements were made over 140 ms *prior* to the opponent striking the ball, *in advance* of pertinent postural information becoming available. They concluded such judgments must be underpinned by the use of contextual information, such as situational probabilities, court positioning and opponent actions and tendencies. In addition, the rate of acquisition of perceptual-cognitive skills from video-based training may be greater when practice conditions include contextual information, such as knowledge of opponent tendencies, although such information appears to limit the use of kinematic information [20]. Further research is required to examine other sources of contextual information and the effect they have on the acquisition and transfer of perceptual-cognitive skills from video-based training to the performance environment.

Researchers have attempted to increase contextual information and task functionality when *testing* anticipation using tasks containing the actions/shots in the sequence they occur in the performance environment [21–27], which is known as a sequential order, as opposed to presenting isolated actions/shots in a random order. Findings demonstrate that presenting the actions/shots in the sequence they occur in the performance environment facilitates more accurate responses when compared to conditions where they are presented in isolation [7]. Furthermore, when the sequence of opponent actions/shots is presented in the order they occur in the performance environment, less cognitive effort is required to make the anticipatory judgments due to the more interpretable information [28]. Researchers have yet to examine the effect of *training* anticipation using video-based tasks in which the opponent actions/shots are presented in the sequence they occur in the performance environment. Such conditions would be expected to improve transfer from the video-based training task to the field setting when compared to conditions in which the same actions/shots are presented in a random sequence [7].

The aim of this study was to examine the retention and transfer benefits of a sequential, compared to random, structure of opponent shots during video-based anticipation training in tennis. Tennis rallies filmed from a first person perspective were displayed life-sized on a screen with the final shot in the rally being occluded. The preceding shots in the rally were presented to two groups in either a sequential or a non-sequential order during training. The non-sequential group was presented with tennis shots in a random order with the assumption that in this condition the main source of information available was the use of advanced postural information. In comparison, the sequential group was presented with the same shots but in the order that they occurred in a rally, allowing for each shot to be presented in “context” and encouraging the use of additional sources of non-kinematic information. The comparison between the two training groups presents a novel attempt to increase task functionality of the practice task and to examine the effect of contextual information on the training of anticipation. Anticipation performance was measured in a pre-test, laboratory retention test, and field-based transfer test. Both groups were expected to improve their anticipation as a result of the training intervention, but the sequential structure was predicted to demonstrate superior retention and transfer, when compared to the non-sequential structure, due to the increased task functionality [6, 8] and additional contextual information [7, 27], but only under retention conditions which matched the practice conditions [11, 20]. Cognitive effort was assessed across practice to provide an insight into the underlying mechanisms of the two structures of practice. It was predicted that the information available to the sequential group is structured in a more interpretable manner as it is more closely aligned to the performance environment when compared to the non-sequential group. Consequently, the predicted learning benefits associated with a sequential structure of practice is hypothesized to coincide with *lower* cognitive effort across practice when compared to a non-sequential order [28].

## Method

### Participants

Participants were 21 intermediate level tennis players who were divided into either a sequential practice (*n* = 11; *M* age = 20.7 years, *SD* = 1.6) or a non-sequential practice group (*n* = 10; *M* age = 20.9 years, *SD* = 1.1). Participants in the two groups were matched by ensuring there were no between-group differences in average start age playing tennis (sequential group: *M* age = 12 years, *SD* = 6; non-sequential group: *M* age = 11 years, *SD* = 6), the average numbers of hours a week they currently played tennis (sequential group: *M* hrs/wk = 4 hrs, *SD* = 3; non-sequential group: *M* hrs/wk = 4 hrs, *SD* = 2), and their average current Lawn Tennis Association (LTA) rating (sequential group: *M* rating = 7.2, *SD* = 0.6; non-sequential group: *M* rating = 8.1, *SD* = 0.8). Separate independent t-tests on each of these variables showed no between-group differences (all *p* > .05). Written informed consent was obtained from the participants prior to taking part and all participants had a right to withdraw at any point. The experiment was conducted in accordance with the 1964 Declaration of Helsinki. Approval was obtained from Liverpool John Moores research ethics committee.

### Task and apparatus

**Fig 1:**
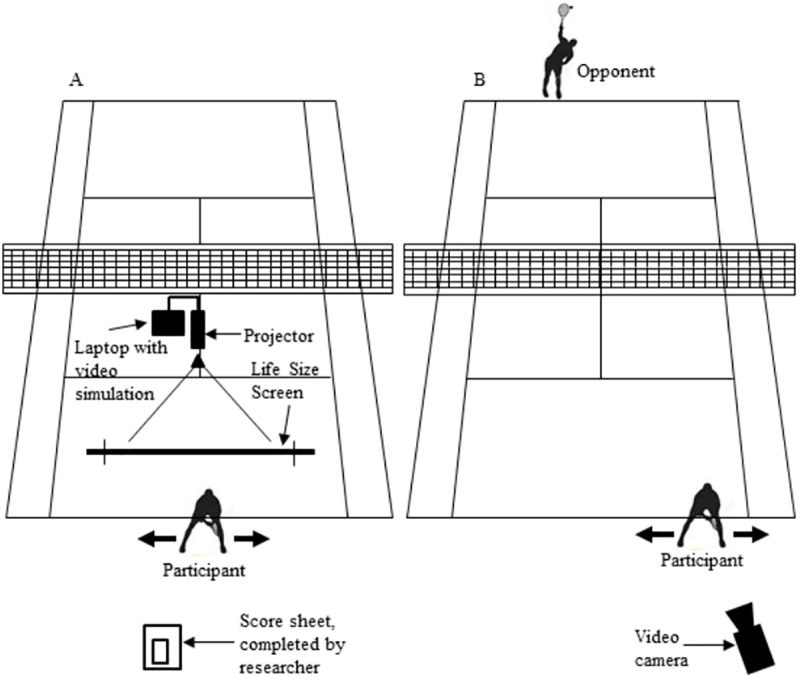
**The experimental setup used in (A) laboratory-based protocol and (B) field-based protocol**.

Fig 1 shows the experimental set up for the video- and field-based protocols.

#### Video-based task

Test films were constructed for the video-based task. Films were constructed in a sequential and non-sequential structure for a pre-test, three practice sessions, and a retention test. In each film, 50% of the final shots were groundstrokes, 33% volleys, and 17% smash shots. Films were shown on a large portable projection screen (2.74 x 3.66 m; Cinefold Projection Sheet, Draper Inc., Spiceland, IN, USA) positioned on a tennis court 4 m from the baseline where the participants stood so that the image of the opponent subtended a vertical visual angle of 4.2°. This matches the 4.3 degree angle that a 1.8-m-tall opponent subtends on an actual tennis court thus replicating the proportions normally experienced in game situations when participants are positioned on the baseline of the court.

The video footage was created by filming an intermediate level tennis player (opponent) executing tennis shots in a rally on court from a first person perspective. A camera (Canon XM-2, Tokyo, Japan) was positioned on the baseline of the court. The opponent was located at the other end of the tennis court and another player (other) was situated behind the camera in order to play return strokes but so that only the opponent appeared in the video footage. Each rally started with a serve from the opponent after which the rally commenced, with a minimum of two other strokes played including the final shot. The importance and prevalence of anticipation in tennis is increased in defensive situations [19], so the opponent played the final shot in each rally from an offensive situation. The other player behind the camera always returned the ball relatively high over the net to the center of the opponent’s side of the court, simulating a defensive shot. In contrast, the opponent played attacking shots to different areas of the court seeking to win the rally.

The footage was edited using Adobe Premier CS5, San Jose, USA. Each trial consisted of video clips of all of the shots from a rally. The start of each trial began with a black screen and the trial number, which appeared for 3 seconds, followed by the initiation of the first video clip. For both groups, the final shot of the rally was the same, and each trial was the same, but the order of the preceding shots was manipulated depending on the group. For the sequential group, the shots preceding the final shot were structured in the manner in which they occurred during the actual rally. Each rally started with the serve and the subsequent shots were in the same order as the rally occurred during filming. For the non-sequential structure group, the preceding shots were in a quasi-random structure so that no rally began with a serve and the penultimate shot was never the same as in the actual rally, making the order of shots random and different to the actual filmed rally. In both practice conditions, all of the shots/clips preceding the final shot contained full ball flight information from the opponent and from the participant side. When the ball flight from the opponent reached the baseline and disappeared off screen behind the camera, the screen went black. When the ball flight of the return shot/clip reappeared on camera, the video started again. In this task, participants held a racket and were instructed that they should move as if they were actually returning the ball in an attempt to simulate match play. These return strokes were used to increase action fidelity in the experiment compared to previous research using video-based tasks [10], rather than as a dependent measure, albeit action fidelity remained somewhat limited by no ball-racket interception.

The final shot in the rally was occluded 80 ms before ball-racket contact and the screen went black. This occlusion point was selected based on previous research that suggests that postural and contextual information will influence anticipatory judgments made at this point [19]. At occlusion of the final stroke, participants simulated a return stroke that indicated their anticipated direction (left/right). Response accuracy was analyzed on the final shot of the rally. Accuracy on the previous shots in the rally was assumed correct, as they had seen full ball flight information. No rallies were repeated across any phase of the experiment.

#### Field-based task

Participants were required to take part in an actual rally on a standard indoor tennis court in which they responded to shots played by an opposing intermediate level tennis player. The opponent was one of the tennis players used for the video footage in the tests and training films. The opponent always served and then the rally commenced. The participant was told to complete rallies against the opponent with the rally ending once the point was won. There were three criteria for a successful rally to be included in the data analysis. These were that it consisted of three or more strokes, the final shot was a winner by the opponent from an offensive situation and not an error by either player, and the final shot landed in the court. The lead experimenter briefed the opponent on the criteria for a “successful” rally and that he was to attempt to win every point using different final shot strokes (groundstroke, volley, smash).

### Procedure

The experiment consisted of pre-tests in the laboratory and field, three practice sessions in the laboratory, and post-tests consisting of a video-based retention test in the laboratory and a field-based transfer test. The pre-tests and practice sessions were completed on the first day and the post-tests were completed seven days later.

#### Pre-tests

Participants completed the video-based task in the laboratory-based pre-test. The films made for the video-based pre-test contained 36 trials. The video-based pre-test was split into two blocks of 18 trials, with one structured in a sequential order and one in a non-sequential order, so as not to favor either group. The video-based pre-test took approximately 15 minutes to complete.

Participants completed the field-based task during the pre-test. For each participant, the first 18 rallies that were deemed suitable were selected for data analysis. A rally was deemed suitable for inclusion when the opponent won the point from an offensive situation, the final shot was by the opponent and was in the court, and the number of shots in the rally was equal to or greater than three strokes. The experimenter used hand notation to record a successful rally based on the criteria detailed above and once 18 successful rallies were completed the field-based test finished (*M* = 25 rallies, *SD* = 5). Participant responses were filmed using a video camera (Canon XM-2, Tokyo, Japan) with wide angled lens and a sampling frequency of 50 Hz [19]. The camera was located behind and to the right of the participant. The moment of opponent racket/ball contact and the movements of the participant were recorded. Decision time (DT) was collected on all shots during a rally, while response accuracy (RA) was collected on only the final shot because all the previous shots were assumed to be accurate as the ball was returned. The field-based pre-test took approximately 30 minutes each to complete.

#### Practice phase

Three video-based practice sessions occurred directly after the laboratory and field pre-tests. Participants completed the video-based task as per the laboratory pre-test, but following their response they received feedback by viewing the full clip of the final shot. No verbal instructions were given regarding the information on screen or participant movements or responses. Participants were not aware of the specific aims of the experiment or conditions. Each practice session consisted of 24 trials and took approximately 15 minutes to complete. Each of the 24 trials was structured in a sequential or in a non-sequential order for that group. The Rating Scale of Mental Effort (RSME) was used to assess the amount of perceived cognitive effort invested in the anticipation task [29]. It was completed twice during a practice block, once after the twelfth trial (i.e. half way) and once at the end of the practice block. An average of the two scores was used as the measure of cognitive effort across the practice phase.

#### Retention tests

The post-tests were the same as the pre-tests, consisting of a video-based retention test in the laboratory and a field-based transfer test.

### Statistical analyses

#### Pre- to retention test

For the video-based task, RA was the primary dependent variable. RA was recorded on only the final shot of the rally/trial. A correct response was when the participant movement responses were to the same side (left, right) as where the ball bounced. To analyze RA in the video-based pre- and retention test, a 2 Group (sequential, non-sequential) x 2 Test (pre, retention) x 2 Practice Condition (sequential, non-sequential) mixed design ANOVA with repeated measures on the last factor was used.

#### Pre- to transfer test

In the field-based tests, RA and DT were the primary dependent variables. As with the video-based protocol, RA was recorded on only the final shot of the rally and was deemed correct when the participant movement responses (left/right) corresponded accurately to where the opponent directed the ball. DT was defined as the time period from ball-racket contact by the opponent to the initiation of movement by the participant (ms). Movement initiation was defined as ‘the first frame where there was an observable and significant lateral motion to the right or left of the racket, the hips, the shoulder or the feet, which was made in order to move to the future location of the next strike’ [19] (p. 822). Movement initiation in tennis usually occurs during or just after a player executes a split-step [30]. When there is no split-step, two distinct behaviors can be found. First, the participant is running without any velocity decrease in which case anticipation is deemed to have started at the beginning of their run. Second, the player is running but there is a decrease in velocity and then an increase, in which case anticipation is deemed to have started at the first frame where there is an increase in velocity. A response initiated prior to ball contact received a negative value.

Footage of the field tests were analyzed using video editing software (Adobe Premier CS5). Inter- and intra-observer reliability measures were obtained for DT by using intraclass correlation techniques [31] on all of the data from two participants, one from each group. For inter-observer reliability, a qualified tennis coach who was provided with the definition given above completed analysis of the same data from two participants. The obtained correlation coefficients for the inter- (.961) and intra-observer (.909) measures demonstrated the data analysis was reliable. To analyze RA and DT in the field pre- and transfer test, separate 2 Group (sequential, non-sequential) x 2 Test (pre, transfer) mixed design ANOVA with repeated measures on the last factor was used. Analysis for DT in the field was not only conducted on the final shot of the rally, but also separately on the average DT of all the shots in the rally to assess overall improvements in anticipation.

#### Practice phase

RA on the final shot of the rally/trial in the video-based task across practice was analyzed using a 2 Group (sequential, non-sequential) x 3 Practice Session (practice 1, practice 2, practice 3) mixed design ANOVA with repeated measures on the last factor. RSME data across practice were analyzed using a 2 Group (sequential, non-sequential) x 3 Practice Session (practice 1, practice 2, practice 3) mixed design ANOVA with repeated measures on the last factor. RSME data were converted into a percentage score for descriptive statistics.

The Bonferroni post hoc procedure was used for all significant within-participant main effects. The Fisher Least Significant Difference (LSD) post hoc procedure was used for all significant interactions. Partial eta squared (*ηp*^*2*^) was used as a measure of effect size (.02 represents a small effect size, 0.5 a medium effect size and 0.8 a large effect size). The alpha level for significance was set at *p* < .05.

## Results

**Fig 2:**
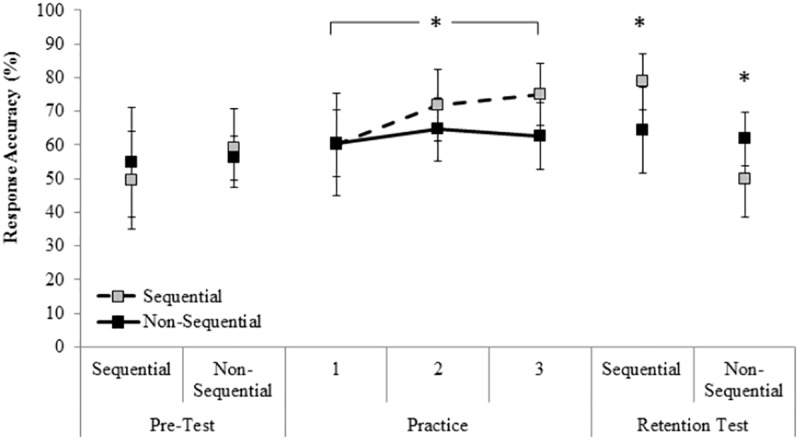
Mean (and SD) response accuracy (%) for the two groups on the laboratory pre-test (sequential, non-sequential), practice 1–3, and the retention test (sequential, non-sequential). *p < .05.

Fig 2 shows mean RA for the two groups on the pre-test (sequential, non-sequential), three practice sessions, and the retention test (sequential, non-sequential).

### Pre- to retention test

The 2 Group x 2 Test x 2 Condition ANOVA on RA in the video-based task revealed a significant main effect for test, *F*(1, 19) = 13.93, *p* < .01, *η*_*p*_^*2*^ = .42. Pairwise comparisons indicated that RA in the retention test (*M* = 64%, *SD* = 8) was significantly higher compared to the pre-test (*M* = 55%, *SD* = 9), p < .01. There was a Test x Condition interaction, *F*(1, 19) = 22.02, *p* < .01, *η*_*p*_^*2*^ = .54, and a three-way Group x Test x Condition interaction, *F*(1, 19) = 14.35, *p* < .01, *η*_*p*_^*2*^ = .43. *Post hoc* analysis indicated no difference in RA between the groups at pre-test in the sequential (sequential group: *M* = 50%, *SD* = 15, non-sequential group: *M* = 55%, *SD* = 16) or non-sequential condition (sequential group: *M* = 59%, *SD* = 12, non-sequential group: *M* = 56%, *SD* = 7). However, in the sequential condition in the retention test, the sequential group (*M* = 79%, *SD* = 8) were significantly more accurate compared to the non-sequential group (*M* = 64%, *SD* = 13), and their own sequential pre-test (*M* = 50%, *SD* = 15). In the non-sequential condition in the retention test, the non-sequential group (*M* = 62%, *SD* = 8) were significantly more accurate when compared to the sequential group (*M* = 50%, *SD* = 12), and their own non-sequential pre-test (*M* = 56%, *SD* = 7). Moreover, for the sequential group, accuracy in the non-sequential retention test was not different to their non-sequential pre-test, whereas for the non-sequential group, accuracy in the sequential retention test was greater compared to their sequential pre-test. There were no other significant main effects or interactions (*p* > .05).

### Pre- to transfer test

**Fig 3:**
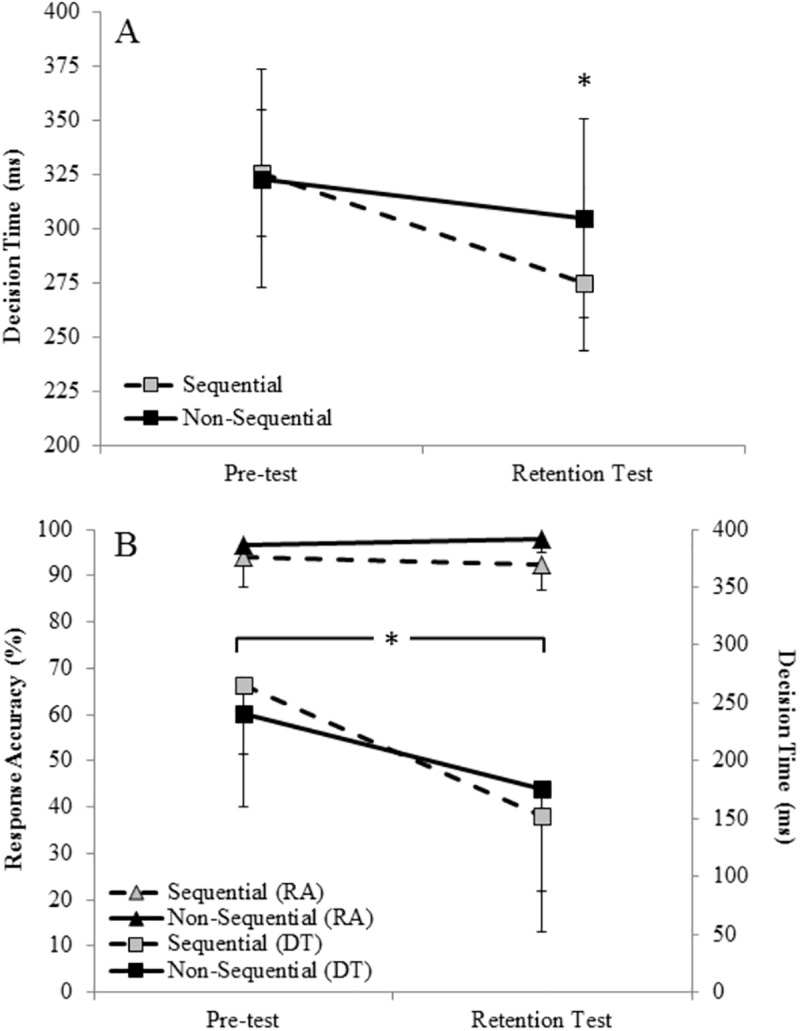
(A) Mean (and SD) decision time (ms) in all shots, and (B) Mean (and SD) decision time (ms) and response accuracy (%) in the final shot, for the two groups (sequential, non-sequential) on the field-based pre-test and the retention test. *p < .05.

Fig 3 shows (a) mean DT in all the shots and (b) mean DT and RA in the final shot, for the two groups (sequential, non-sequential) on the pre-test and the transfer test.

#### Response accuracy

The 2 Group x 2 Test ANOVA revealed a significant group main effect, *F*(1, 19) = 10.16, *p* = .01, *η*_*p*_^*2*^ = .35. Pairwise comparisons indicated that overall RA for the non-sequential group was significantly higher compared to the sequential group. This difference was found in the pre-test and in the transfer test. There were no other significant main effects or interactions (*p* > .05).

#### Decision time

For all shots, the 2 Group x 2 Test ANOVA revealed no significant group main effect, *F*(1, 19) = .78, *p* = .39, *η*_*p*_^*2*^ = .04. However, there was a significant main effect for test, *F*(1, 19) = 19.40, *p* < .01, *η*_*p*_^*2*^ = .51. Pairwise comparisons indicated that DT in the transfer test (*M* = 290ms, *SD* = 41) was significantly faster compared to the pre-test (*M* = 324ms, *SD* = 39). There was also a Group x Test interaction, *F*(1, 19) = 4.27, *p* = .05, *η*_*p*_^*2*^ = .18. *Post hoc* analysis indicated that the sequential group significantly reduced their DT from pre- (*M* = 325ms, *SD* = 29) to transfer test (*M* = 275ms, *SD* = 31), whereas the non-sequential group showed no significant improvements from pre- (*M* = 323ms, *SD* = 50) to retention (*M* = 305ms, *SD* = 46).

For the final shot, the 2 Group x 2 Test ANOVA revealed no significant group main effect, *F*(1, 19) < .01, *p* = .98, *η*_*p*_^*2*^ < .01. However, there was a significant main effect for test, *F*(1, 19) = 19.92, *p* < .01, *η*_*p*_^*2*^ = .51. Pairwise comparisons indicated that DT in the transfer test (*M* = 164ms, *SD* = 93) was significantly faster compared with the pre-test (*M* = 253ms, *SD* = 70). There were no other significant main effects or interactions (*p* > .05).

### Practice phase

#### Response accuracy

The 2 Group x 3 Practice Session ANOVA revealed a group main effect for RA, *F*(1, 19) = 7.77, *p* = .01, *η*_*p*_^*2*^ = .29. Pairwise comparisons indicated that RA in the sequential group (*M* = 69%, *SD* = 13) was significantly higher compared to the non-sequential group (*M* = 63%, *SD* = 10). There was also a significant session main effect, *F*(2, 38) = 3.35, *p* = .05, *η*_*p*_^*2*^ = .15. *Post hoc* analysis indicated that RA in the first practice session (*M* = 60%, *SD* = 13) was significantly lower compared to the second (*M* = 68%, *SD* = 11) and third practice session (*M* = 69%, *SD* = 11), between which there was no difference in RA. There was no interaction between Group and Session (*p* > .05).

#### Rating scale of mental effort

The 2 Group x 3 Practice ANOVA revealed a significant group main effect, *F*(1, 19) = 6.66, *p* = .02, *η*_*p*_^*2*^ = .26. Pairwise comparisons indicated that cognitive effort was significantly higher during practice for the non-sequential group (*M* = 57%, *SD* = 10) compared to the sequential group (*M* = 46%, *SD* = 10). There was no significant main effect for session or interaction (*p* > .05).

## Discussion

In the current study, video-based training using footage of tennis rallies was used to examine the effect of practice structure on the retention and transfer of anticipatory behavior in tennis. Participants viewed the rallies in either a sequential or a non-sequential order during training. Anticipation performance was measured over a pre-test, three practice sessions, a laboratory retention test, and a field-based transfer test. In the laboratory retention test, both groups were expected to improve anticipation performance compared to the pre-test. However, it was predicted that the sequential structure group would demonstrate superior RA in the retention test due to the increased task functionality and the additional information available during practice, when compared to the non-sequential group [6, 7]. Similarly, in the field-based transfer test it was expected that both groups would reduce DT following practice, but the sequential group would demonstrate faster DT compared to the non-sequential group. It was predicted that cognitive effort across practice would be lower for the sequential group, as the information available in that condition was presented in a more interpretable manner, when compared to a non-sequential structure of practice [28].

As predicted, both groups improved RA significantly from the pre- to retention test, providing support for previous research showing video-based training leads to perceptual-cognitive skill acquisition [14–17]. In addition, the sequential group demonstrated greater improvements in accuracy between the pre-test and sequential retention test when compared to the non-sequential group [21–27]. These findings indicate that a sequential structure of practice in which actions/shots are presented in the order they occur in the performance environment is a potential method to improve performance by increasing contextual information and task functionality when training anticipation in video-based experiments [6, 7]. However, findings in the non-sequential retention condition differed to that in the sequential retention condition. The sequential group reverted to their performance levels in the pre-test in the non-sequential retention condition. In contrast, RA for the non-sequential group was not different in the sequential and non-sequential retention condition. The contextual information available during training seems to limit the use of advanced postural information, reducing anticipation performance for the sequential group when the contextual information was not present in the non-sequential retention test [20]. The non-sequential group, on the other hand, is assumed to have learned during training to identify advanced postural information to improve accuracy, as no other contextual information was available during practice. Postural information was available in *both* of the retention conditions, so the non-sequential group was more accurate in both retention conditions, when compared to their pre-test. A trade-off occurred between the benefits of contextual information during practice when the condition replicated the retention test, and the cost of this condition when such information was not present in the other retention test, supporting the *specificity of practice* hypothesis [11]. Given that Triolet et al. [19] demonstrated the use of advanced postural information *and* the processing of contextual information underpinned anticipation during elite level tennis matches, our data supports the propositions of Cañal-Bruland and Mann [7] that researchers should broaden the scope of research to consider contextual information. As expected, the sequential group had lower cognitive effort during practice when compared to the non-sequential group, due to the information being presented in a more interpretable manner [28]. The sequential structure of practice likely increased task functionality, compared to the non-sequential structure, exposing that group to constraints that more closely matched those of the performance environment when compared to previous research.

In the field-based transfer test, RA on the final shot of each rally did not differ between the pre- and retention test. As in previous studies with field-based transfer tests, RA was over 90% for both groups in the pre- and retention test, suggesting a ‘ceiling effect’ [15–17]. The full vision conditions in the field-based tests lead to high RA because a rich and detailed visual display is available (e.g., ball flight), as well as other key environmental information, such as auditory and proprioceptive feedback from producing an interceptive action [4]. In contrast, video-based experimental tasks occlude vision of ball flight usually at ball-racket contact. In field-based protocols, DT often differentiates training from control groups [15–17]. Therefore, it was expected that both groups would reduce DT following practice, but DT would be further reduced for the sequential compared to non-sequential group. As predicted, the sequential group had significantly reduced overall DT in the field-based transfer test compared to the non-sequential group and the pre-test. Findings demonstrate that a sequential structure of practice during video-based training leads to transfer benefits for anticipation in the field. As discussed previously, this effect may be due to increased task functionality in practice as the shots were structured in a more interpretable manner and presented additional sources of information that facilitated greater transfer to the performance environment. However, DT on the final shot showed that both groups reduced DT from the pre- to transfer test, but there were no between-group differences. The final shots were all attacking winners compared to the previous neutral rally strokes. Kinematic differences between attacking and neutral strokes may have meant that the advanced pick up of postural information became more salient and pertinent on the final shot resulting in a lack of between-group differences. In contrast, in neutral situations where the opponent is attempting to gain control of the rally and create an unfavorable situation, contextual information (e.g., opponent tendencies) may hold greater importance, although future research is required to examine this. Findings highlight the importance of training individuals to recognize both kinematic and contextual sources of information during video-based training.

A limitation of the current findings is that participants were intermediate level tennis players and findings may not generalize to novice athletes who do not have the domain-specific knowledge to interpret the contextual information arising from shots ordered in the sequence they occur in the performance environment [26]. In future, researchers should consider how practice structure interacts with skill level [4, 28]. Moreover, researchers focusing on elite athletes could examine the role of other contextual factors on performance in an effort to further increase task functionality and the specificity of practice, such as opponent playing characteristics or the effects of different court surfaces (for example, see [32]). While the notion of *specificity* is already fundamental to perceptual-motor skill acquisition [11], the current findings suggest that further research is required to investigate this concept in perceptual-cognitive skills training (see also, [33]). The current research is also limited by the fact that video-based tasks do not include an interceptive action or the exact movements that occur in the performance environment, which may or may not be an important component of anticipatory behavior [34]. Research is required to examine further the effects of action fidelity in anticipation testing and training experiments. Finally, research is required to investigate the importance to anticipation of the interaction between kinematic and contextual information, taking into account types of situations and their evolving time frames.

In summary, we provide a novel insight into the design of video-based tasks for the training of anticipation by examining the effect of sequential and non-sequential structures of practice on retention and transfer performance [4]. The sequential structure was proposed to increase task functionality during practice [6], such that information was organized in a more interpretable manner [28] and included additional sources of contextual information that are critical for performance [7]. Our novel findings demonstrated that training with a structure of practice in which actions/shots are presented in the order they occur in the performance environment resulted in significantly superior retention performance compared to a non-sequential structure of practice, but only when the retention condition replicated the training conditions [11]. In the non-sequential retention condition, RA in the sequential group reverted to pre-test levels, whereas RA for the non-sequential group was not different between the sequential and non-sequential retention condition. The non-sequential group may have learnt to recognize advanced postural information during practice, and as this information was available in *both* of the retention conditions, anticipation performance did not differ between conditions. In contrast, the contextual information available during training for the sequential group may have limited their use of advanced postural information, reducing their RA when the contextual information was not present in the non-sequential retention test [20]. Transfer of learning was found from the video-based training to the field-based performance environment for both practice groups, with a sequential structure of practice resulting in significantly reduced overall DT compared to the non-sequential group. Findings highlight the benefits of designing video-based practice tasks in a more representative manner by including key contextual sources of information [7, 18].
